# Association between opioid use and survival in advanced non small cell lung cancer patients treated with immune checkpoint inhibitors

**DOI:** 10.1038/s41598-025-15684-4

**Published:** 2025-08-18

**Authors:** Luigi Liguori, Stefano Pepe, Valentina Pagliara, Rosario De Feo, Alessandro Ottaiano, Francesco Perri, Giovanna Polcaro, Valeria Conti, Cristina R. Ferrone, Francesco Sabbatino, Marco Cascella

**Affiliations:** 1 Department of Medicine, Surgery and Dentistry “Scuola Medica Salernitana”, University of Salerno, 84081 Baronissi, Italy; 2 Division of Innovative Therapies for Abdominal Metastases, Istituto Nazionale Tumori IRCCS Fondazione G. Pascale, 80131 Naples, Italy; 3 Medical and Experimental Head and Neck Oncology Unit, Istituto Nazionale Tumori IRCCS Fondazione G. Pascale, 80131 Naples, Italy; 4 Department of Surgery, Cedars-Sinai Medical Center, Los Angeles, CA 90048 USA

**Keywords:** Biomarker, ICI, NSCLC, Opioid, PD-1, Real-world, Cancer, Biomarkers, Health care, Medical research, Oncology

## Abstract

Cancer-related pain is a frequent challenge among non-small cell lung (NSCLC) cancer patients, particularly for those with advanced disease and/or bone metastases. Opioids are the mainstay of treatment for moderate to severe cancer-related pain. However, emerging lines of evidence suggest that concomitant opioid use may be associated with poor survival outcomes in advanced NSCLC patients treated with immune checkpoint inhibitors (ICIs). We analyzed the impact of concomitant opioid use on survival outcomes of advanced NSCLC patients treated with ICIs. Correlations between baseline clinical-pathological characteristics and survival outcomes were assessed using log-rank tests while multivariate survival analyses were performed using the Cox proportional hazards model. Among patients treated with ICI as monotherapy and those treated with ICI as second or subsequent lines of treatment, concomitant opioid use was correlated with decreased progression-free survival (PFS) (*p* = 0.0460 and *p* = 0.0490) and overall survival (OS) (*p* = 0.0380 and *p* = 0.0230) in univariate analyses. However, in multivariate analyses, concomitant opioid use was not independently correlated with survival outcomes. Instead, ECOG PS ≥ 2 and bone metastases emerged as strong predictors of decreased PFS and OS. Despite limitations, our findings highlight that concomitant opioid use does not independently correlate with poor survival outcomes in this setting of patients.

## Introduction

Cancer-related pain is a common and debilitating condition, affecting up to 70% of cancer patients in advanced and terminal stages of disease^1^. Despite opioids remaining the first-line therapy for managing moderate to severe cancer-related pain^2^, emerging lines of evidence have raised concerns regarding the potential impact of concomitant opioid use on survival outcomes in cancer patients. For instance, Boland et al. reported a negative association between concomitant opioid use and survival rates, though the causal relationship remains unclear and likely influenced by various confounding factors^3^.

In parallel, immune checkpoint inhibitors (ICIs) targeting programmed cell death 1 (PD-1) and its ligand 1 (PD-L1) have revolutionized the treatment landscape for many types of cancers including non-small cell lung (NSCLC)^4^. ICIs enhance the immune system’s ability to recognize and attack tumor cells^5^, significantly improving survival rates in selected patient populations as compared to standard therapies^4^. However, the potential impact of concomitant opioid use on survival outcomes of advanced NSCLC patients treated with ICIs remains uncertain. Some studies suggested that concomitant opioid use may be correlated with poor survival outcomes, through mechanisms involving immunosuppression or alterations of the tumor microenvironment^6^. However, the robustness of these results is limited by their retrospective nature as well as by confounding variables and methodological constraints^7^.

In a recent study, we investigated the potential correlation between clinical-pathological characteristics and survival outcomes in advanced NSCLC patients treated with ICI^8^, which demonstrated a potential correlation between concomitant opioid use and poor survival outcomes^8^. To validate these preliminary results, we performed further analyses of specific patient subpopulations, while minimizing confounding variables. The objective was to better understand the potential impact of concomitant opioid use on survival outcomes in advanced NSCLC patients treated with ICIs.

## Results

### Clinical-pathological characteristics and clinical outcomes of specific patient subpopulations

Of the patients with advanced NSCLC treated with ICI-based immunotherapy^8^, the following specific patient subpopulations were analyzed: (i) patients treated with ICI as monotherapy, (ii) patients treated with the combination of platinum-based chemotherapy and ICI; (iii) patients treated with ICI as first-line treatment; (iv) patients treated with ICI as second or subsequent lines of treatment; (v) patients younger than 65 years treated with ICI; and (vi) patients aged 65 years or older treated with ICI.

The results highlight that most patients across all subpopulations were male, with females representing a smaller proportion. Most patients had an Eastern Cooperative Oncology Group (ECOG) Performance Status (PS) of 0 or 1, though higher PS scores (2–3) were more common in older patients or those receiving subsequent treatments. PD-L1 tumor proportion scores varied, with higher expression (≥ 50%) more common in patients treated with monotherapy. Lymph nodes were the most frequent metastatic site, followed by bone and central nervous system (CNS) metastases. Treatment regimens ranged from ICI monotherapy to combination therapies with platinum-based chemotherapy. Moreover, CR rates were low across subpopulations, while PR and SD contributed to ORR and DCR that varied between 8.9–24.5% and 41–61.2%, respectively. Median PFS ranged from 3.90 months in subsequent treatment groups to 8.37 months in patients receiving platinum-based chemotherapy plus ICI, while OS followed a similar trend, with younger patients and those on combination therapies achieving better survival outcomes. Baseline clinical-pathological characteristics and outcomes of these subpopulations are reported in Tables 1 and 2, respectively.

**Table 1:** Baseline clinical-pathological characteristics of specific patient subpopulations.

Baseline clinical-pathological characteristics	Patients treated with ICI as monotherapy	Patients treated with the combination of platinum-based chemotherapy and ICI	Patients treated with ICI as first-line treatment	Patients treated with ICI as second or subsequent lines of treatment	Patients younger than 65 years treated with ICI	Patients aged 65 years or older treated with ICI
Sex						
Male	63 (78.7%)	37 (75.5%)	55 (75.3%)	45 (80.4%)	33 (73.3%)	67 (79.8%)
Female	17 (21.3%)	12 (24.5%)	18 (24.7%)	11 (19.6%)	12 (26.7%)	17 (20.2%)
ECOG PS						
0	25 (31.3%)	22 (44.9%)	33 (45.2%)	14 (25.0%)	24 (53.3%)	23 (27.4%)
1	37 (46.2%)	21 (42.9%)	29 (39.7%)	29 (51.8%)	15 (33.3%)	43 (51.2%)
2	14 (17.5%)	5 (10.2%)	9 (12.3%)	10 (17.9%)	4 (8.9%)	15 (17.9%)
3	4 (5.0%)	1 (2.0%)	2 (2.7%)	3 (5.4%)	2 (4.4%)	3 (3.6%)
Smoking status						
Never smoker	6 (7.5%)	8 (16.3%)	12 (16.4%)	2 (3.6%)	4 (8.9%)	10 (11.9%)
Previous smoker	50 (62.5%)	27 (55.1%)	40 (54.8%)	37 (66.1%)	26 (57.8%)	51 (60.7%)
Current smoker	24 (30.0%)	14 (28.6%)	21 (28.8%)	17 (30.4%)	15 (33.3%)	23 (27.4%)
Comorbidities						
Hypertension	49 (61.3%)	22 (44.9%)	37 (50.7%)	34 (60.7%)	17 (37.8%)	54 (64.3%)
Dyslipidemia	28 (35.0%)	9 (18.4%)	22 (30.1%)	15 (26.8%)	5 (11.1%)	32 (38.1%)
Diabetes	19 (23.8%)	7 (14.3%)	12 (16.4%)	14 (25.0%)	3 (6.7%)	23 (27.4%)
Chronic obstructive pulmonary disease	16 (20.0%)	6 (12.2%)	11 (15.1%)	11 (19.6%)	6 (13.3%)	16 (19.0%)
Heart failure	8 (10.0%)	2 (4.1%)	7 (9.6%)	3 (5.4%)	1 (2.2%)	9 (10.7%)
Depressive disorder	2 (2.5%)	2 (4.1%)	2 (2.7%)	2 (3.6%)	2 (4.4%)	2 (2.4%)
Chronic renal failure	1 (1.3%)	0 (0.0%)	0 (0.0%)	1 (1.8%)	0 (0.0%)	1 (1.2%)
Previous cancer						
None	73 (91.2%)	42 (85.7%)	66 (90.4%)	49 (87.5%)	40 (88.9%)	75 (89.3%)
Prostate cancer	6 (7.5%)	7 (14.3%)	7 (9.6%)	6 (10.7%)	5 (11.1%)	8 (9.5%)
Breast cancer	1 (1.3%)	0 (0.0%)	0 (0.0%)	1 (1.8%)	0 (0.0%)	1 (1.2%)
Concomitant medications						
Anticoagulants	14 (17.5%)	10 (20.4%)	16 (21.9%)	8 (14.3%)	7 (15.6%)	17 (20.2%)
Antiplatelet drugs	20 (25.0%)	4 (8.2%)	7 (9.6%)	17 (30.4%)	4 (8.9%)	20 (23.8%)
Antihypertensives	47 (58.8%)	22 (44.9%)	36 (49.3%)	33 (58.9%)	16 (35.6%)	53 (63.1%)
Oral hypoglycemic drugs	15 (18.8%)	4 (8.2%)	10 (13.7%)	9 (16.1%)	1 (2.2%)	18 (21.4%)
Statins	21 (26.3%)	11(22.4%)	19 (26.0%)	13 (23.2%)	6 (13.3%)	26 (31.0%)
Antidepressants	2 (2.5%)	4 (8.2%)	4 (5.5%)	2 (3.6%)	2 (4.4%)	4 (4.8%)
Opioids	21 (26.3%)	12(24.5%)	19 (26.0%)	14 (25.0%)	16 (35.6%)	17 (20.2%)
Benzodiazepines	2 (2.5%)	1 (2.0%)	1 (1.4%)	3 (5.4%)	2 (4.4%)	1 (1.2%)
Antineuralgics	8 (10.0%)	6 (12.2%)	7 (9.6%)	7 (12.5%)	8 (17.8%)	6 (7.1%)
Antipsychotics	1 (1.3%)	1 (2.0%)	1 (1.4%)	1 (1.8%)	1 (2.2%)	1 (1.2%)
Low dose aspirin	29 (36.4%)	12 (24.5%)	20 (27.4%)	21 (37.5%)	7 (15.6%)	34 (40.5%)
Baseline prednisone equivalent dose						
≤ 10 mg/day	57 (71.3%)	22 (44.9%)	43 (58.9%)	36 (64.3%)	24 (53.3%)	55 (65.5%)
> 10 mg/day	23 (28.7%)	27 (55.1%)	30 (41.1%)	20 (35.7%)	21 (46.7%)	29 (34.5%)
PD-L1 TPS						
NA	30 (37.5%)	1 (2.0%)	1 (1.4%)	30 (53.6%)	8 (17.8%)	23 (27.4%)
< 1%	15 (18.8%)	22 (44.9%)	23 (31.5%)	14 (25.0%)	13 (28.9%)	24 (28.6%)
≥ 1% < 50%	4 (5.0%)	26 (53.1%)	26 (35.6%)	4 (7.1%)	15 (33.3%)	15 (17.8%)
≥ 50%	31 (38.7%)	0 (0.0%)	23 (31.5%)	8 (14.3%)	9 (20.0%)	22 (26.2%)
Histology						
Adenocarcinoma	41 (51.1%)	39 (79.6%)	49 (67.1%)	31 (55.4%)	35 (77.8%)	45 (53.6%)
Squamous cell carcinoma	36 (45.0%)	8 (16.3%)	21 (28.8%)	23 (41.1%)	9 (20.0%)	35 (41.6%)
Large cell carcinoma	2 (2.6%)	2 (4.1%)	2 (2.7%)	2 (3.6%)	1 (2.2%)	3 (3.6%)
Sarcomatoid carcinoma	1 (1.3%)	0 (0.0%)	1 (1.4%)	0 (0.0%)	0 (0.0%)	1 (1.2%)
Sites of metastasis						
Lymph node	71 (89.9%)	41 (83.7%)	63 (86.3%)	49 (89.1%)	42 (93.3%)	70 (84.3%)
Lung	53 (67.1%)	31 (63.3%)	43 (58.9%)	41 (74.5%)	30 (66.7%)	54 (65.1%)
Bone	24 (30.4%)	14 (28.6%)	22 (30.1%)	16 (29.1%)	16 (35.6%)	22 (26.5%)
Central nervous system	18 (22.8%)	10 (20.4%)	11 (15.1%)	17 (30.9%)	14 (31.1%)	14 (16.9%)
Adrenal gland	15 (19.0%)	8 (16.3%)	15 (20.5%)	8 (14.5%)	9 (20.0%)	14 (16.9%)
Liver	10 (12.7%)	5 (10.2%)	7 (9.6%)	8 (14.5%)	4 (8.9%)	11 (13.3%)
Skin	4 (5.1%)	2 (4.1%)	4 (5.5%)	2 (3.6%)	2 (4.4%)	4 (4.8%)
Type of therapy						
Nivolumab	45 (56.3%)	0 (0.0%)	0 (0.0%)	45 (80.4%)	12 (26.7%)	33 (39.3%)
Pembrolizumab	26 (32.5%)	0 (0.0%)	24 (32.9%)	2 (3.6%)	9 (20.0%)	17 (20.3%)
Atezolizumab	9 (11.2%)	0 (0.0%)	0 (0.0%)	9 (16.0%)	2 (4.4%)	7 (8.3%)
Carboplatin-pemetrexed-pembrolizumab	0 (0.0%)	37 (75.5%)	37 (50.7%)	0 (0.0%)	18 (40.0%)	19 (22.6%)
Carboplatin-nab paclitaxel-pembrolizumab	0 (0.0%)	7 (14.3%)	7 (9.6%)	0 (0.0%)	1 (2.2%)	6 (7.1%)
Carboplatin-pemetrexed-ipilimumab-nivolumab	0 (0.0%)	5 (10.2%)	5 (6.8%)	0 (0.0%)	3 (6.7%)	2 (2.4%)

ECOG: Eastern Cooperative Oncology Group, ICI: Immune checkpoint inhibitor, NA: Not available, PD-L1: Programmed death ligand 1, PS: Performance status, TPS: Tumor proportion score.

**Table 2:** Clinical outcomes of specific patient subpopulations.

Clinical outcomes	Patients treated with ICI as monotherapy	Patients treated with the combination of platinum-based chemotherapy and ICI	Patients treated with ICI as first-line treatment	Patients treated with ICI as second or subsequent lines of treatment	Patients younger than 65 years treated with ICI	Patients aged 65 years or older treated with ICI
Best response						
CR	1 (1.2%)	0 (0.0%)	1 (1.4%)	0 (0.0%)	0 (0.0%)	1 (1.2%)
PR	10 (12.5%)	10 (20.4%)	15 (20.5%)	5 (8.9%)	11 (24.5%)	9 (10.8%)
SD	22 (27.5%)	20 (40.8%)	24 (32.9%)	18 (32.1%)	15 (33.3%)	27 (32.1%)
PD	37 (46.3%)	4 (8.2%)	16 (21.9%)	25 (44.7%)	14 (31.1%)	27 (32.1%)
NA	10 (12.5%)	15 (30.6%)	17 (23.3%)	8 (14.3%)	5 (11.1%)	20 (23.8%)
ORR	15.0%	20.4%	21.9%	8.9%	24.5%	12%
DCR	41.2%	61.2%	54.8%	41.0%	57.8%	44.1%
Median PFS	4.43 months (95% CI 3.40–7.17 months)	8.37 months (95% CI 5.23–17.70 months)	5.87 months (95% CI 4.90–15.30 months)	3.90 months (95% CI 3.07–8.10 months)	6.87 months (95% CI 5.17–18.70 months)	4.90 months (95% CI 3.43–9.53 months)
Median OS	8.17 months (95% CI 5.17–12.70 months)	11.80 months (95% CI 5.33–18.50 months)	11.80 months (95% CI 5.33–18.70 months)	8.17 months (95% CI 4.47–12.10 months)	10.80 months (95% CI 6.83–46.80 months)	7.83 months (95% CI 4.37–12.70 months)

CI: confidence interval, CR: complete response, DCR: disease control rate, ICI: Immune checkpoint inhibitor, ORR: objective response rate, OS: overall survival, PD: progressive disease, PFS: progression-free survival, PR: partial response, SD: stable disease.

### Correlations between concomitant opioid use and survival outcomes

Subsequently, correlations between the concomitant opioid use and survival outcomes were explored in each of the subpopulations analyzed.

**Fig. 1:**
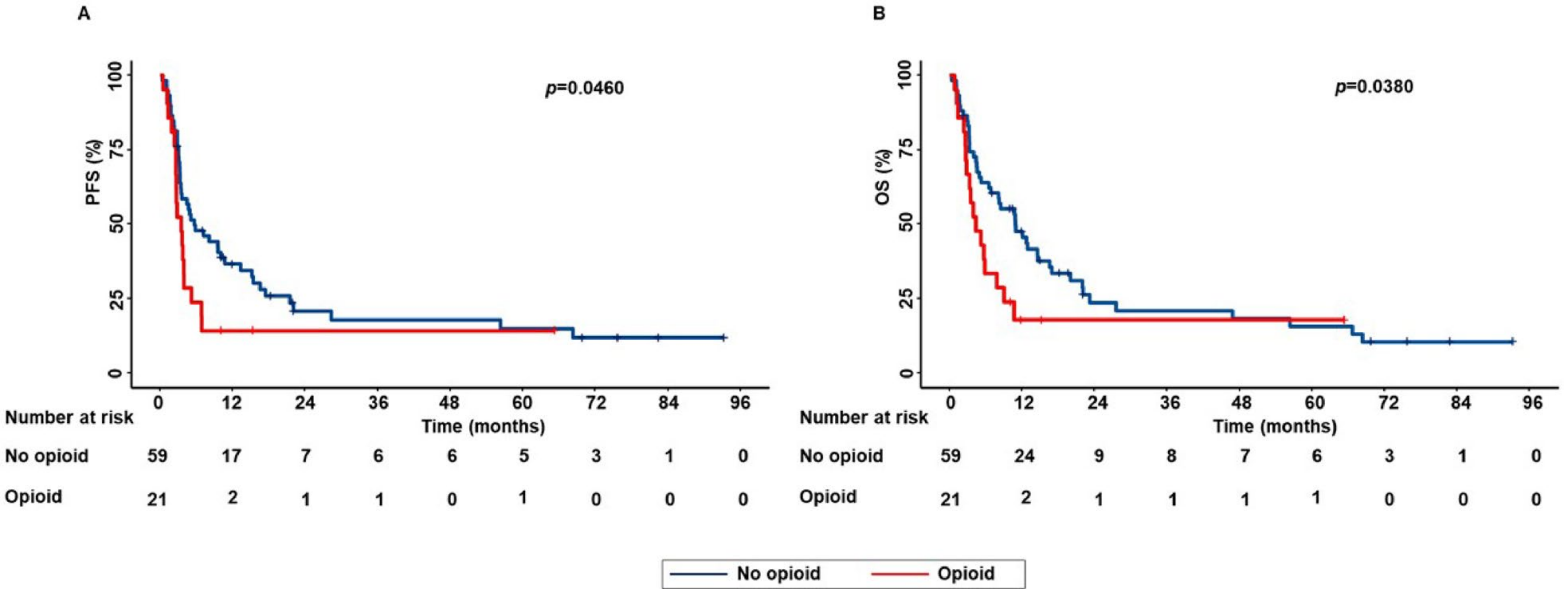
Correlation between concomitant opioid use and survival outcomes in advanced NSCLC patients treated with ICI as monotherapy. PFS (**A**) and OS (**B**) of patients were stratified based on concomitant opioid use. PFS and OS were compared using the Kaplan–Meier method. Differences in patients’ survival were analyzed using a log-rang test. *p* < 0.05 was considered statistically significant.

In the subpopulation of patients treated with ICI as monotherapy, concomitant opioid use was significantly correlated with decreased PFS (*p* = 0.0460) and OS (*p* = 0.0380) (Fig. 1).

**Fig. 2:**
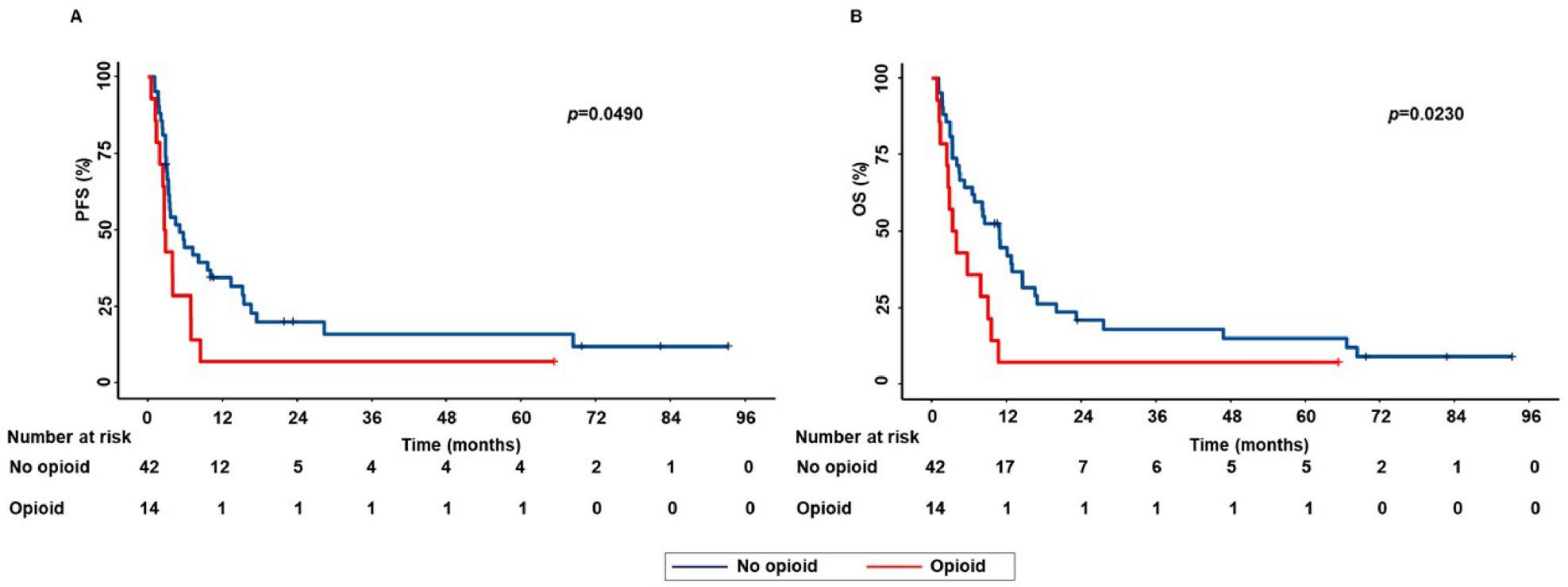
Correlation between concomitant opioid use and clinical outcomes in advanced NSCLC patients treated with ICI as second or subsequent lines of treatment. PFS (**A**) and OS (**B**) of patients were stratified based on concomitant opioid use. PFS and OS were compared using the Kaplan–Meier method. Differences in patients’ survival were analyzed using a log-rang test. *p* < 0.05 was considered statistically significant.

On the same line, in the subpopulation of patients treated with ICI as second or subsequent lines of treatment, concomitant opioid use was significantly correlated with decreased PFS (*p* = 0.0490) and OS (*p* = 0.0230) (Fig. 2).

Conversely, in the other subpopulations (patients treated with the combination of platinum-based chemotherapy and ICI, patients treated with ICI as first-line treatment, patients younger than 65 years treated with ICI and patients aged 65 years or older treated with ICI) no significant correlation between concomitant opioid use and both PFS and OS was found (Table 3).

**Table 3:** Opioid-outcome correlations in specific patient subpopulations.

Subpopulation	*P* value for PFS	*p* value for OS
Patients treated with the combination of platinum-based chemotherapy and ICI	*p* = 0.1720	*p* = 0.1270
Patients treated with ICI as first-line treatment	*p* = 0.0910	*p* = 0.0990
Patients younger than 65 years treated with ICI	*p* = 0.0637	*p* = 0.0530
Patients aged 65 years or older treated with ICI	*p* = 0.0720	*p* = 0.0410

ICI: Immune checkpoint inhibitor, OS: overall survival, PFS: progression-free survival.

### Correlations between baseline clinical-pathological characteristics and survival outcomes

To validate the observed correlations between concomitant opioid use and survival outcomes, we investigated potential correlations between other baseline clinical-pathological characteristics and survival outcomes in the subpopulations where the concomitant opioid use was significantly correlated with both PFS and OS.

In the subpopulation of patients treated with ICI as monotherapy, the concomitant use of steroids, bone metastases, and ECOG PS were significantly correlated with survival outcomes.

**Fig. 3:**
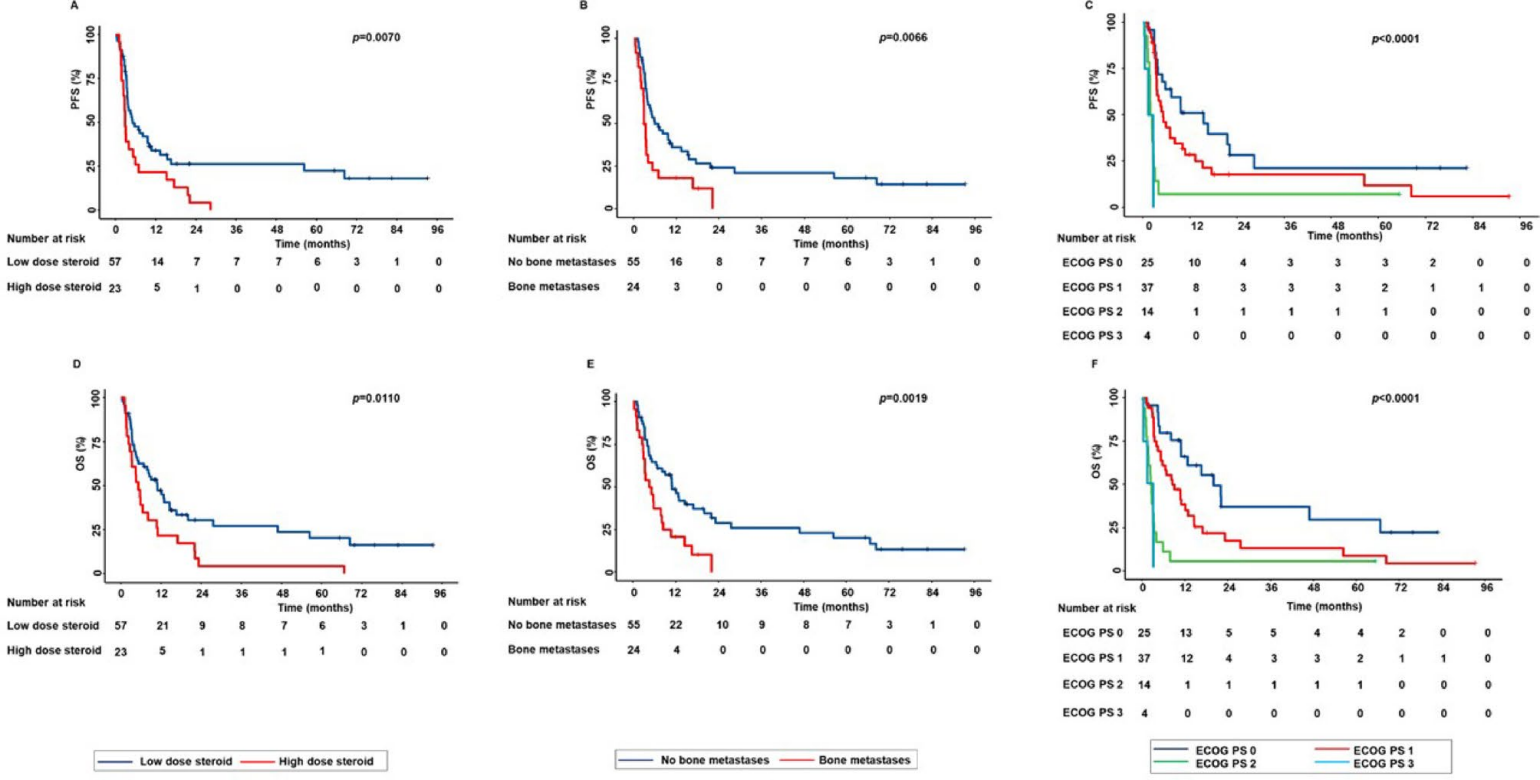
Correlations between baseline clinical-pathological characteristics and survival outcomes in advanced NSCLC patients treated with ICI as monotherapy. The concomitant use of steroids (more than 10 mg/day of prednisone equivalent dose), the presence of bone metastases, and ECOG PS 2–3 were correlated with decreased PFS (**A**–**C**) and OS (**D**–**F**). PFS and OS were compared using the Kaplan–Meier method. Differences in patients’ survival were analyzed using a log-rang test. *p* < 0.05 was considered statistically significant.

More in detail, (i) concomitant use of a high dose of steroids (more than 10 mg/day of prednisone equivalent dose) was significantly correlated with decreased PFS (*p* = 0.0070) and OS (*p* = 0.0110) than concomitant use of a low dose of steroids (less than 10 mg/day of prednisone equivalent dose) (Fig. 3A–D); (ii) presence of bone metastases was significantly correlated with decreased PFS (*p* = 0.0066) and OS (*p* = 0.0019) than the absence of bone metastases (Fig. 3B–E); and (iii) patients with ECOG PS 2–3 were correlated with decreased PFS (*p* < 0.0001) and OS (*p* < 0.0001) than those with ECOG PS 0 or 1 (Fig. 3C–F).

**Fig. 4:**
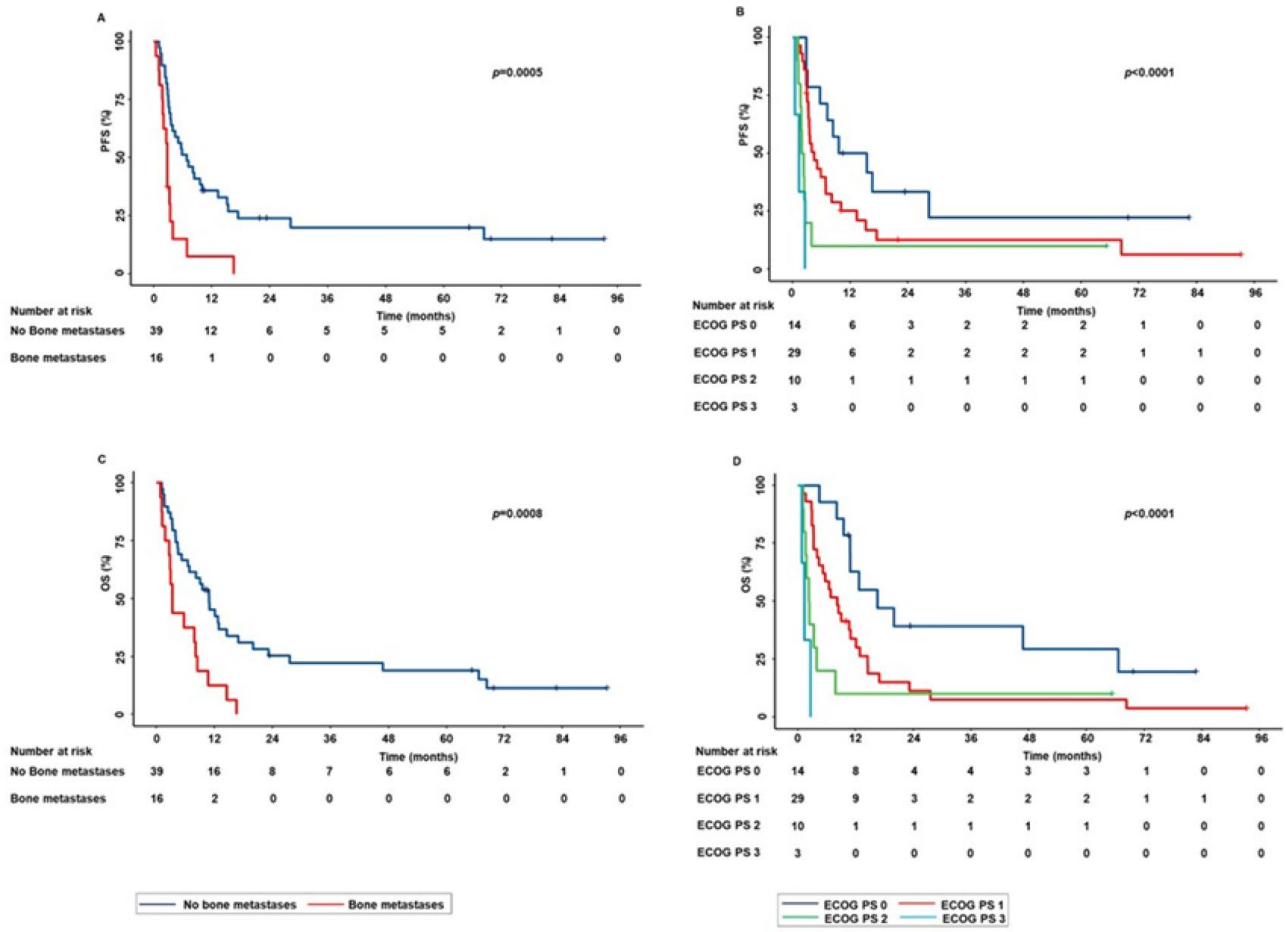
Correlations between baseline clinical-pathological characteristics and survival outcomes in advanced NSCLC patients treated with ICI as second or subsequent lines of treatment. The presence of bone metastases and ECOG PS 2–3 were correlated with decreased PFS (**A**,**B**) and OS (**C**,**D**). PFS and OS were compared using the Kaplan–Meier method. Differences in patients’ survival were analyzed using a log-rang test. *p* < 0.05 was considered statistically significant.

On the other hand, in the subpopulation of patients treated with ICI as second or subsequent lines of treatment, the bone metastases and ECOG PS were significantly correlated with survival outcomes. More in detail, (i) presence of bone metastases was significantly correlated with decreased PFS (*p* = 0.0005) and OS (*p* = 0.0008) than the absence of bone metastases (Fig. 4A–C) (ii) patients with ECOG PS 2–3 were correlated with decreased PFS (*p* < 0.0001) and OS (*p* < 0.0001) than those with ECOG PS 0–1 (Fig. 4B–D). In contrast, the concomitant use of steroids was not significantly correlated with PFS (*p* = 0.0860) and OS (*p* = 0.01070).

### Validation of the correlations between concomitant opioid use and survival outcomes in multivariate analyses

To validate the correlations between the concomitant opioid use and survival outcomes, multivariate analyses were performed.

**Fig. 5:**
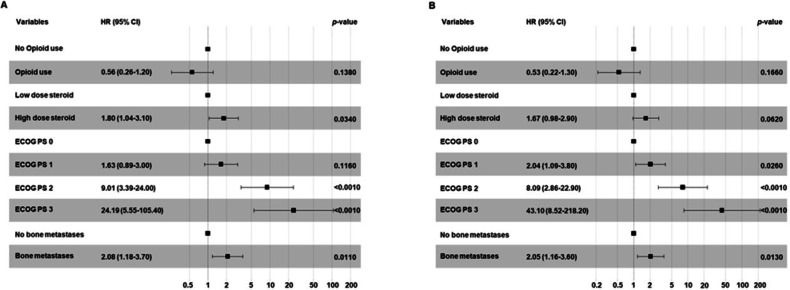
Multivariate analyses testing the correlations between baseline clinical-pathological characteristics and PFS (**A**) or OS (**B**) in advanced NSCLC patients treated with ICI as monotherapy. Multivariate survival analyses were performed using the Cox proportional hazards model.

In the subpopulation of patients treated with ICI as monotherapy, the concomitant opioid use was not significantly correlated with PFS (*p* = 0.1380) and OS (*p* = 0.1660). Conversely, patients with ECOG PS 2–3 and those with bone metastases were correlated with both decreased PFS (*p* < 0.0010 and *p* = 0.0110) and OS (*p* < 0.0010 and *p* = 0.0130). The concomitant use of a high dose of steroids was significantly correlated with decreased PFS (*p* = 0.0340) while no significant correlation between the concomitant use of a high dose of steroids and OS was found (*p* = 0.0620) (Fig. 5).

**Fig. 6:**
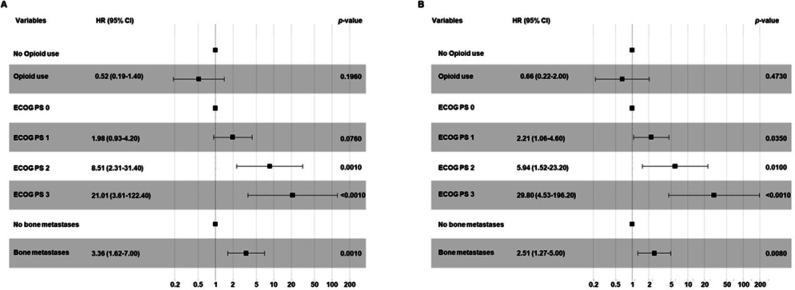
Multivariate analysis testing the correlations between baseline clinical-pathological characteristics and PFS (**A**) or OS (**B**) in advanced NSCLC patients treated with ICI as second or subsequent treatment. Multivariate survival analyses were performed using the Cox proportional hazards model.

On the other hand, in the subpopulation of patients treated with ICI as second or subsequent lines of treatment, the concomitant opioid use was not significantly correlated with PFS (*p* = 0.1960) and OS (*p* = 0.4730). Conversely, patients with ECOG PS 2–3 as well as those with bone metastases were correlated with both decreased PFS (*p* < 0.0010 and *p* = 0.0010) and OS (*p* < 0.0010 and *p* = 0.0080) (Fig. 6).

## Discussion

In this analysis of a retrospective real-world study of patients with advanced NSCLC, we evaluated the impact of concomitant opioid use and other baseline clinical-pathological characteristics on survival outcomes. We demonstrated that the concomitant opioid use does not independently correlate with poor survival outcomes across the different subpopulations. We stratified patients by treatment type (ICI monotherapy vs combination of platinum-based chemotherapy and ICI), line of treatment (first vs second or subsequent) and age (less than 65 years old vs 65 years or older), as these variables are well-known to influence the survival outcomes of cancer patients^9,10^. Specifically, in four of the six patient subpopulations analyzed, concomitant opioid use was not significantly correlated with both PFS and OS. However, in the remaining two subpopulations (patients treated with ICI as monotherapy and those treated with ICI as second or subsequent lines of treatment) concomitant opioid use was significantly correlated with decreased survival outcomes in univariate analyses. A possible explanation for the observed association between concomitant opioid use and poorer survival outcomes is that opioid prescriptions may act as a surrogate marker of more advanced disease, such as the presence of bone metastases and/or poor ECOG PS. Indeed, the correlation between concomitant opioid and survival outcomes was not confirmed in multivariate analyses, which accounted for other significant variables such as the concomitant use of steroids, ECOG PS, and bone metastases. Our findings suggest that the observed impact on survival outcomes is more likely due to the presence of bone metastases and/or ECOG PS 2–3, rather than the concomitant opioid use. This finding is supported by the significant correlations between concomitant opioid use and bone metastases (*p* = 0.0449 and *p* = 0.0460) as well as ECOG PS (*p* = 0.0003 and *p* = 0.0002) in both patient subpopulations. As a result, the poor outcome of concomitant opioid use is likely to reflect the presence of bone metastases and poor ECOG PS in those patients who consume opioids. These results are consistent with other studies demonstrating that bone metastases and poor ECOG PS significantly impact survival outcomes^11–13^. In addition, emerging preclinical studies have investigated the potential biological mechanisms underlying these correlations. In particular, the presence of bone metastases and poor ECOG PS are linked to i) a state of systemic inflammation marked by elevated levels of immunosuppressive cytokines (such as IL-6, IL-10, TNF-α) and ii) an immunosuppressive tumor microenvironment characterized by reduced CD8 + T cell levels alongside increased infiltration of myeloid-derived suppressor cells and T reg cells^14,15^. These factors have already been implicated in ICI resistance and decreased survival^16^.

In the past few years, other studies have investigated the potential impact of concomitant opioid use on survival outcomes in advanced NSCLC patients treated with ICIs. For instance, Hong et al. have recently reported a negative correlation between concomitant opioid use and prognosis across various types of cancer including NSCLC. However, the authors did not include ECOG PS or bone metastases in their multivariate analyses, which may have confounded their results^17^. In contrast, Young et al. have demonstrated that concomitant opioid use remained significant even when accounting for variables like ECOG PS and bone metastases^18^. However, their study included a broader population of patients treated with both ICI as monotherapy and those treated with the combination of platinum-based chemotherapy and ICI, which could introduce additional biases.

To address this issue, we analyzed different subpopulations based on ICI treatment regimens and found that the significant correlation between concomitant opioid use and survival outcomes was not present in a multivariate analysis even when we compared patients treated with ICI monotherapy and ICI in combination with platinum-based chemotherapy. As a result, concomitant opioid use does not have a clinically relevant impact on survival outcomes in advanced NSCLC, regardless of ICI treatment regimens, lines of treatment, or age of the patients.

In this study, we also evaluated the potential impact of concomitant medications such as steroids. In the subpopulation of patients treated with ICI as monotherapy, multivariate analyses demonstrated that concomitant use of high dose steroids was significantly correlated with decreased PFS while no significant correlation with OS was observed. This discrepancy may be explained by various factors, including differences in the type and number of subsequent therapies. On the other hand, in the subpopulation of patients treated with a combination of platinum-based chemotherapy and ICI, the concomitant use of high dose steroids did not affect the survival outcomes in the univariate analysis. One possible explanation is that platinum-based chemotherapy may counteract the potential detrimental effect of high dose steroids on ICI. Additionally, in the subpopulation of patients treated with ICI as a second or subsequent line of treatment, no correlation between concomitant steroid use and survival outcome was identified. To date, many other studies have investigated the potential impact of steroids on survival outcomes in advanced NSCLC patients treated with ICI. However, the results are mixed and no consensus has yet been reached^19,20^. Emerging preclinical studies have investigated the potential biological mechanisms underlying the impact of steroid use in patients treated with ICI. For instance, Maeda et al. demonstrated that the administration of steroids can promote the exhaustion of T cells by upregulating their PD-1 expression^21^, a key mechanism of ICI resistance^16^. Furthermore, steroids can also impair antigen presentation by downregulating human leucocyte antigen (HLA) class I on cancer cells, and promoting intrinsic tumor resistance to ICIs^22^. These effects are consistent with results demonstrating that steroids suppress cytotoxic T cell activity, inhibit pro-inflammatory cytokine release, and elevate T reg cell counts, all contributing to tumor extrinsic ICI resistance^23^. No further correlation between other concomitant medications or their associated comorbidities and survival outcomes was reported in our analysis.

The retrospective design and single-center nature are important limitations of this study. Additionally, we did not assess the dose of opioids, which may have a role in this context. Other studies have already investigated whether different opioid doses (high versus low dose) potentially influence survival outcomes, with mixed results^18,24^. Additionally, cancer-related pain often necessitated escalating doses of opioids as well as further doses of opioids for the treatment of breakthrough pain making it difficult to define a clear cut-off for testing the correlations between different opioid doses and survival outcomes. Lastly, the sample sizes of the specific subpopulations analyzed were relatively small, which may limit the generalizability of our findings.

## Materials and methods

### Study design and population

Clinical data of patients with confirmed advanced (stage IV) NSCLC treated with ICI from January 2016 to December 2023 at “San Giovanni di Dio e Ruggi D’Aragona” University Hospital, Italy, was retrospectively reviewed. The study was approved by the local ethics committee (prot./SCCE n.85275) and was carried out following the Declaration of Helsinki and its amendments. All patients signed informed consent for clinical-pathological data acquisition.

The following patient subpopulations were analyzed: (i) patients treated with ICI as monotherapy, (ii) patients treated with the combination of platinum-based chemotherapy and ICI; (iii) patients treated with ICI as first-line treatment; (iv) patients treated with ICI as second or subsequent lines of treatment; (v) patients younger than 65 years treated with ICI; and (vi) patients aged 65 years or older treated with ICI.

Inclusion criteria encompassed: (i) age ≥ 18 years; (ii) histologically confirmed diagnosis of advanced NSCLC; (iii) treatment with ICI as monotherapy or in combination with platinum-based chemotherapy. Exclusion criteria encompassed: (i) presence of “oncogene-addicted” tumor alterations in Epidermal Growth Factor Receptor (*EGFR*), Anaplastic Lymphoma Kinase (*ALK*), c-ros oncogene 1 (*ROS1*), V-Raf Murine Sarcoma Viral Oncogene Homolog B (*BRAF*), Mesenchymal-epithelial transition factor (*MET*), REarranged during Transfection (*RET*) and Neurotrophic Tropomyosin Receptor Kinases (*NTRK*); (ii) presence of active autoimmune disease.

Evaluation of ALK, BRAF, EGFR, MET, NTRK, RET, and ROS1 tumor alterations was performed on tumor samples (when available) or liquid biopsy according to national pathology guidelines. PD-L1 was evaluated on tumor samples as clinically indicated when tumor tissue was available and reported as tumor proportion score (TPS) according to European Society for Medical Oncology (ESMO) guidelines.

Clinical-pathological characteristics included age, sex, ECOG PS, smoking status, comorbidities, previous cancer, concomitant medications, baseline prednisone equivalent dose, PD-L1 TPS, histologic subtypes, specific sites of metastasis and type of immunotherapy.

According to Italian guidelines, patients received one of the following ICI-based treatments: (i) atezolizumab or nivolumab after the failure of platinum-based chemotherapy, regardless PD-L1 TPS; (ii) pembrolizumab after the failure of platinum-based chemotherapy for PD-L1 TPS ≥ 1%; (iii) combination of platinum-based chemotherapy and pembrolizumab or combination of platinum-based chemotherapy and nivolumab and ipilimumab as first-line for PD-L1 TPS < 50%; (iv) pembrolizumab as first-line for PD-L1 TPS ≥ 50%.

Immune-related adverse events (irAEs) were defined as adverse events displaying a certain, likely, or possible correlation with ICI according to Common Terminology Criteria for Adverse Events (CTCAE) v 4.0^25^. Radiographic imaging was performed every two months, according to clinical practice. Radiographic response was determined according to Response Evaluation Criteria in Solid Tumours version 1.1 (RECIST v1.1)^26^ and reported as complete response (CR), partial response (PR), stable disease (SD), and progression disease (PD).

The proportion of patients with CR or PR accounted for the objective response rate (ORR) while that of patients with CR or PR or SD the disease control rate (DCR). Progression-free survival (PFS) was defined as the time from the start of the treatment to the first documented PD or death by any cause. Overall survival (OS) was defined from the start of the treatment to death by any cause or last follow-up date.

### Statistical analysis

Data was collected using Microsoft Excel. Statistical analyses were performed using STATA v13 software released by StataCorp LP (College Station, TX, USA).

Categorical variables were expressed as frequencies and percentages. PFS and OS were calculated using the Kaplan–Meier method. Correlations between baseline clinical-pathological characteristics and survival outcomes (PFS and OS) were performed using the log-rank test. Multivariate survival analyses were performed using the Cox proportional hazards model. The difference between groups was considered significant when the *p*-value was < 0.05.

## Conclusion

Despite the limitations of our real-world investigation, we conclude that concomitant opioid use is not significantly correlated with survival outcomes in advanced NSCLC treated with ICI. Additionally, our study highlights that patients with ECOG PS 2–3 and those with bone metastases had a higher opioid use and were characterized by worse overall survival outcomes. However, given conflicting evidence regarding the impact of concomitant opioid use on survival outcomes as well as its increasing use in cancer pain management, further larger prospective studies are needed to elucidate whether the concomitant opioid use influences the survival outcomes of advanced NSCLC patients treated with ICI. Furthermore, novel investigations should also explore the potential mechanisms underlying the correlation between opioid use and alteration of the tumor immune response. Finally, these studies should always consider other key baseline clinical-pathological characteristics such as ECOG PS and bone metastases, to avoid confounding results.

## Data Availability

The datasets generated during and/or analyzed during the current study are available from the corresponding author on reasonable request.
